# Babesiosis among Elderly Medicare Beneficiaries, United States, 2006–2008

**DOI:** 10.3201/eid1801.110305

**Published:** 2012-01

**Authors:** Mikhail Menis, Steven A. Anderson, Hector S. Izurieta, Sanjai Kumar, Dale R. Burwen, Jonathan Gibbs, Garner Kropp, Tugce Erten, Thomas E. MaCurdy, Christopher M. Worrall, Jeffrey A. Kelman, Mark O. Walderhaug

**Affiliations:** Food and Drug Administration, Rockville, Maryland, USA (M. Menis, S.A. Anderson, H.S. Izurieta, S. Kumar, D.R. Burwen, M.O. Walderhaug);; Acumen LLC, Burlingame, California, USA (J. Gibbs, G. Kropp, T. Erten, T.E. MaCurdy);; Centers for Medicare & Medicaid Services, Baltimore, Maryland, USA (C.M. Worrall, J.A. Kelman)

**Keywords:** vector-borne infections, zoonoses, babesiosis, parasites, ICD-9-CM code, elderly, protozoan, Medicare, administrative databases, United States

## Abstract

We used administrative databases to assess babesiosis among elderly persons in the United States by year, sex, age, race, state of residence, and diagnosis months during 2006–2008. The highest babesiosis rates were in Connecticut, Rhode Island, New York, and Massachusetts, and findings suggested babesiosis expansion to other states.

Human babesiosis is a zoonotic disease caused by intraerythrocytic protozoan parasites of *Babesia* species. In the United States, *Babesia microti* is the primary etiologic agent of human babesiosis and is usually transmitted through the bite of *Ixodes scapularis*, the principal tick vector for this species (*1**–**3*). Human *B. microti* infections are regional, endemic to Northeastern (Connecticut, Rhode Island, Massachusetts, New York, New Jersey) and Midwestern (Minnesota, Wisconsin) states, and the geographic range is believed to be expanding (*1**,**2**,**4**,**5*). Babesiosis is characteristically seasonal, with peak transmission from May through September (*1**,**2**,**6*). In younger persons, babesiosis is more likely to be a mild or asymptomatic disease that may persist for months or even years undetected (*1**,**2**,**7**,**8*). Elderly, splenectomized, and other immunocompromised persons tend to be symptomatic (e.g., fever, chills, fatigue) and at risk for complications, including hemolytic anemia, acute respiratory failure, renal failure, and death (*1**–**3**,**9*).

Recently, there has been an increase in the number of reported clinical and transfusion-transmitted babesiosis cases in the United States (*1**,**2**,**10*). Efforts to mitigate transfusion-transmitted babesiosis risk include development of donor screening tests and testing strategies. Initiatives on babesiosis encompassed addition of *Babesia* spp. infections to the list of nationally notifiable diseases in 2011, a Food and Drug Administration sponsored workshop (*1*), a Blood Products Advisory Committee Meeting (*10*), and creation of the AABB *Babesia* Task Force. Because elderly persons are one of the most vulnerable at-risk populations and there are no published nationwide studies on babesiosis in that group, we used Centers for Medicare & Medicaid Services (Baltimore, MD, USA) administrative databases to assess babesiosis among elderly Medicare beneficiaries in the United States during 2006–2008.

## The Study

We used 100% inpatient, outpatient, skilled nursing facility, and carrier standard analytical as well as Medicare enrollment files for calendar years 2006–2008 to assess babesiosis among elderly Medicare beneficiaries ages >65 years of age. Standard analytical files are generated to capture medical services rendered and patient diagnoses, and enrollment files help to ascertain coverage eligibility. To be eligible for the study, beneficiaries had to be continuously enrolled in Medicare fee-for-service Parts A and B for >365 days before and including the latest month of continuous enrollment in the calendar year. We identified likely new babesiosis cases on the basis of the first recording of the International Classification of Diseases, 9th Revision, Clinical Modification diagnosis code 088.82 during the calendar year, with no recorded babesiosis infection in the preceding 365 days.

We assessed annual babesiosis rates by estimating the number of cases recorded per 100,000 beneficiaries per calendar year, overall and by sex, age, race, and state of residence. Seasonal occurrence was analyzed by using the number of cases in each month and number of beneficiaries continuously enrolled in Medicare fee-for-service within 365 days of each month. Cases were assigned age on the basis of diagnosis date, and persons without babesiosis had age assessed at the beginning of the latest enrollment month in the year. Beneficiaries with babesiosis were excluded from denominators of subsequent calendar years or diagnosis months. We performed χ^2^ tests comparing babesiosis rates by using Epi Info version 3.5.1 (wwwn.cdc.gov/epiinfo). We conducted this institutional review board-approved study in coordination with the Centers for Medicare & Medicaid Services and within the SafeRx Project.

Among 27,278,865, 26,381,435, and 25,908,122 elderly Medicare beneficiaries in 2006, 2007, and 2008, respectively, there were 985 (3.6/100,000), 851 (3.2/100,000), and 1,223 (4.7/100,000) babesiosis cases based on International Classification of Diseases, 9th Revision, Clinical Modification diagnosis code (Table 1). Annual babesiosis rates (per 100,000 beneficiaries) among the white elderly were 4.0, 3.6, and 5.2, and among nonwhite elderly the rates were 0.6, 0.9, and 1.4 for 2006–2008, respectively. Rate comparisons for whites versus nonwhites showed significant differences (p<0.0001) for each year. Babesiosis rates were significantly (p<0.001) higher for men versus women and younger elderly women (ages 65–84 years) versus older elderly women (>85 years old) (Table 1). The Figure displays highest babesiosis rates in July and August of each year, with 74.4% of cases diagnosed in May through October.

**Table 1:** Number of babesiosis cases and rates by sex and age among elderly Medicare beneficiaries, United States, 2006–2008

Sex and age group, y	No. beneficiaries with babesiosis (rate/100,000*)				Total no. enrolled beneficiaries		
	2006	2007	2008		2006	2007	2008
F							
>65	509 (3)	441 (3)	647 (4)		15,919,094	15,356,954	15,032,121
65–74	283 (4)	240 (4)	365 (6)		6,794,195	6,547,813	6,487,120
75–84	171 (3)	153 (3)	214 (4)		6,075,497	5,781,146	5,527,531
>85	55 (2)	48 (2)	68 (2)		3,049,402	3,027,995	3,017,470
M							
>65	476 (4)	410 (4)	576 (5)		11,359,771	11,024,481	10,876,001
65–74	248 (4)	195 (3)	313 (6)		5,792,828	5,601,691	5,563,925
75–84	179 (4)	165 (4)	209 (5)		4,211,721	4,051,649	3,921,203
>85	49 (4)	50 (4)	54 (4)		1,355,222	1,371,141	1,390,873
Both							
>65	985 (4)	851 (3)	1,223 (5)		27,278,865	26,381,435	25,908,122
65–74	531 (4)	435 (4)	678 (6)		12,587,023	12,149,504	12,051,045
75–84	350 (3)	318 (3)	423 (4)		10,287,218	9,832,795	9,448,734
>85	104 (2)	98 (2)	122 (3)		4,404,624	4,399,136	4,408,343

*Babesiosis rates were rounded to the nearest whole number.

**Figure:**
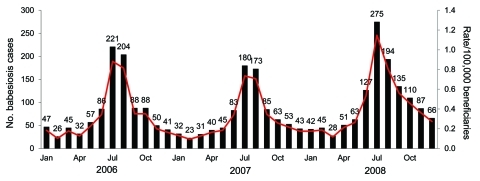
Number of babesiosis cases (black bars) and rates (red line) by month of diagnosis among elderly Medicare beneficiaries, United States, 2006–2008.

Babesiosis varied by state, with the top 10 states and District of Columbia accounting for 834 (84.7%), 709 (83.3%), 1,024 (83.7%) of all cases in 2006–2008, respectively (Table 2). Connecticut, Rhode Island, New York, and Massachusetts had the highest babesiosis rates among the elderly (Table 2). Babesiosis cases were also recorded in other states, including California, Florida, Texas, Pennsylvania, Minnesota, and Wisconsin, with those 6 states accounting for 9.8%, 10.8%, and 12.3% of all cases in 2006, 2007, and 2008, respectively (data not shown).

**Table 2:** Highest babesiosis rates among elderly Medicare beneficiaries, by state, United States, 2006–2008*

State or district	No. beneficiaries with babesiosis (rate/100,000†)				Total no. enrolled beneficiaries		
	2006	2007	2008		2006	2007	2008
US total	985 (4)	851 (3)	1,223 (5)		27,278,865	26,381,435	25,908,122
Connecticut	166 (42)	168 (44)	183 (50)		394,431	381,453	364,449
Rhode Island	34 (45)	13 (18)	30 (41)		74,951	73,387	72,430
New York	395 (24)	268 (17)	379 (25)		1,621,816	1,559,031	1,508,401
Massachusetts	76 (13)	128 (22)	185 (32)		596,349	590,318	586,278
Maryland	43 (8)	34 (7)	61 (12)		508,445	510,573	512,902
New Jersey	80 (9)	59 (7)	98 (11)		887,588	884,107	885,544
District of Columbia	3 (7)	1 (2)	3 (7)		44,419	43,843	42,986
Virginia	25 (3)	29 (4)	59 (8)		749,256	723,406	718,145
Maine	7 (4)	4 (2)	10 (6)		180,030	179,797	176,233
New Hampshire	3 (2)	4 (3)	9 (6)		149,654	151,120	150,567
Delaware	2 (2)	1 (1)	7 (7)		102,197	103,937	105,359

*States include District of Columbia. States are shown in descending order of average babesiosis rate during the 3-year period. †Babesiosis rates were rounded to the nearest whole number.

## Conclusions

We report a national population-based study of babesiosis among the US elderly, which used large administrative databases. The study found variations in the number of babesiosis cases by year, state, race, sex, age, and diagnosis month. Overall, our 3-year study suggests that there were more cases of babesiosis in 2008 compared with previous years. Northeastern and Mid-Atlantic States accounted for most newly diagnosed cases among the US elderly, with state-specific rates up to 10× higher than national annual rates. Our results show highest babesiosis rates in known babesiosis-endemic states of Connecticut, Rhode Island, New York, and Massachusetts and suggest possible expansion of human babesiosis to Maryland, Virginia, and other states. Human encroachment into tick and deer habitat, growth of deer population, climatic effects, and travel to disease-endemic areas may be responsible for variations in number of babesiosis cases and spread of the infection to non–disease-endemic states (*1**–**3**,**9**,**11*).

Our findings show babesiosis trends similar to surveillance results in Rhode Island and New York State over the same period, with fewer cases reported in 2007, and more in 2008 (*12**,**13*). Data from the Connecticut Department of Public Health demonstrate broad annual variation in numbers of reported babesiosis cases in 2000–2008, with an increasing trend over time (*14*). Our finding of higher babesiosis rates among men versus women and among younger elderly women versus older elderly women are generally consistent with state surveillance data (*12**,**15*). Similarly to the literature (*1**,**2**,**6**,**12**,**15*), our study shows that most babesiosis cases are diagnosed during May through October. These findings are likely related to life cycle and activity of the tick vector and to activity of human and other mammalian hosts (*1**,**2**,**9*).

Study limitations are related to the use of administrative databases and include difficulty in identifying incident versus prevalent cases, possible misdiagnosis, and lack of clinical detail for diagnosis verification and transfusion-transmitted babesiosis case identification, as well as inability to differentiate *Babesia* species. Medical record review is needed to address above-mentioned limitations. Choosing a different continuous enrollment period could produce slightly different rates. Although Medicare data do not provide population-wide information on babesiosis among persons <65 years of age, younger persons are more likely to remain asymptomatic and less likely to get a diagnosis (*1**,**2**,**7*).

Our nationwide large medical database study is an additional tool to better understand regional, seasonal, and other babesiosis transmission patterns, by year and demographic characteristics, among the US elderly. Because the elderly are also known to use the majority of transfused blood, studies are needed to evaluate transfusion-transmitted babesiosis in this group. Overall, our study suggests that large administrative databases can be useful in assessing emerging infections in the United States.
